# The Molecular Organization of Human cGMP Specific Phosphodiesterase 6 (PDE6): Structural Implications of Somatic Mutations in Cancer and Retinitis Pigmentosa

**DOI:** 10.1016/j.csbj.2019.03.004

**Published:** 2019-03-06

**Authors:** Arooma Maryam, Sundeep Chaitanya Vedithi, Rana Rehan Khalid, Ali F. Alsulami, Pedro Henrique Monteiro Torres, Abdul Rauf Siddiqi, Tom L. Blundell

**Affiliations:** a Department of Biosciences, COMSATS University Islamabad (CUI), Park Road, Islamabad, Pakistan; b Department of Biochemistry, University of Cambridge, 80 Tennis Court Rd, Cambridge CB2 1GA, UK

**Keywords:** Somatic mutations, Cancer, Retinitis pigmentosa, Nitric oxide (NO), Cyclic guanosine monophosphate (cGMP), Phosphodiesterase 6 (PDE6)

## Abstract

In the cyclic guanosine monophosphate (cGMP) signaling pathway, phosphodiesterase 6 (PDE6) maintains a critical balance of the intracellular concentration of cGMP by catalyzing it to 5′ guanosine monophosphate (5′-GMP). To gain insight into the mechanistic impacts of the PDE6 somatic mutations that are implicated in cancer and retinitis pigmentosa, we first defined the structure and organization of the human PDE6 heterodimer using computational comparative modelling. Each subunit of PDE6αβ possesses three domains connected through long α-helices. The heterodimer model indicates that the two chains are likely related by a pseudo two-fold axis. The N-terminal region of each subunit is comprised of two allosteric cGMP-binding domains (Gaf-A & Gaf-B), oriented in the same way and interacting with the catalytic domain present at the C-terminal in a way that would allow the allosteric cGMP-binding domains to influence catalytic activity. Subsequently, we applied an integrated knowledge-driven in silico mutation analysis approach to understand the structural and functional implications of experimentally identified mutations that cause various cancers and retinitis pigmentosa, as well as computational saturation mutagenesis of the dimer interface and cGMP-binding residues of both Gaf-A, and the catalytic domains. We studied the impact of mutations on the stability of PDE6αβ structure, subunit-interfaces and Gaf-cGMP interactions. Further, we discussed the changes in interatomic interactions of mutations that are destabilizing in Gaf-A (R93L, V141 M, F162 L), catalytic domain (D600N, F742 L, F776 L) and at the dimer interface (F426A, F248G, F424 N). This study establishes a possible link of change in PDE6αβ structural stability to the experimentally observed disease phenotypes.

## Graphical Abstract

**Figure:**
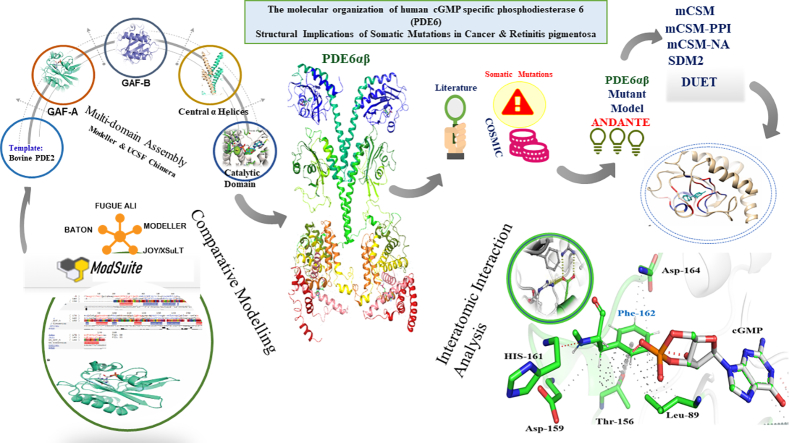

## 1 Introduction

In living organisms, synchronized cellular communication among integrated signaling circuits is necessary to ensure proper cellular function. Accurate signal transduction of extracellular cues to interior of the cells is coordinated between multiple compartmentalized intracellular signaling pathways through the recruitment of a distinct subset of sensing molecules [51,59]. So far 3000 signaling proteins and approximately 15 distinct second messenger molecules are known in mammals [24,35]. In the late 1980s, nitric oxide (NO) was discovered as the first diffusible primary signaling molecule that binds and activates soluble guanylate cyclase (sGC) to produce 3′, 5′cyclic guanosine monophosphate (cGMP) from guanosine triphosphate (GTP). Cyclic guanosine monophosphate is a vital secondary messenger molecule that elicits specific biological effects by activating various effector proteins through cGMP specific protein kinase (PKG). While cyclic nucleotide phosphodiesterases (PDEs) hydrolyze cyclic GMP to 5′-GMP [9,27]. Phosphodiesterases act at the crossroad of multitude of intertwined compartmentalized intracellular signaling pathways where they are negative regulators of the concentration of cGMP and operate to dampen its downstream signaling circuitry in specific subcellular compartments [20]. In the cGMP signaling pathway, homeostatic regulation of signals propagating within the various interlinked intracellular compartments rely heavily on the dynamic organization of sGC, PKG and PDE, multimeric signaling complexes as well as it depending on the critical balance of primary and secondary diffusible messenger molecules [1].

The phosphodiesterase superfamily is encoded by 21 genes that are categorized into 11 structurally homologous isozyme subfamilies based on their substrate specificity [1,20]. PDE6, known as photoreceptor phosphodiesterase, is the only member of the enzymes superfamily that predominantly expresses in the cytosol of retinal photoreceptor cells. Localization of PDE6 has also been reported in lungs, malpighian tubules of kidneys and pineal gland [26]. Recently, a study reported subcellular localization of retina-restricted PDE6 in the perinuclear region of endothelial cells and suggested its significant role in compartmentalized cGMP signaling pathways of endothelium [8]. In pineal glands, PDE6 regulates light induced melatonin production. Through melatonin production PDE6 indirectly regulates melanin production, sleep-wake cycles, biological rhythms and cell repair mechanisms in response to stress and disease [41,56,62]. PDE6, like the other five closely related cGMP specific PDEs possess tandemly arranged GAF domains at the N-terminal that controls dimer formation and act as allosteric binding site of cGMP molecules [20]. Furthermore, the rod photoreceptor specific PDE6 is unique among its enzyme family members as it is comprised of two different catalytic subunits, an α subunit encoded by PDE6A gene and a β subunit encoded by PDE6B. Both these subunits helps in lowering the cGMP concentration by hydrolyzing it into GTP [8,15].

Recent advances of in-depth DNA and RNA sequencing analyses of cancer genomes are resulting into a growing repertoire of somatic mutations in signaling proteins [13]. Abnormalities in the cGMP signaling pathway have recently identified to amplify proliferative signaling in human cancer [5]. Elevation of cGMP concentration, as a result of defects and/or aberrant expression of photoreceptor specific PDE6, leads to hyper-activation of PKG isoforms, which in turn contributes to the progression of malignant melanoma in skin and breast cancer cells [7,39] and various kinds of cancer pathologies in lungs, stomach, endometrium and as well as in familial retinitis pigmentosa [14,16,23,32,55]. The Cancer Genome Atlas (TCGA) project has given insight into recurrent mutant genes in colorectal cancer (CRC) through large-scale genome sequencing. They investigated somatic mutations and chromosomal trasnlsocations in various members of WNT, RAS-MAPK, PI3K, TGF-β, P53 and DNA mismatch repair pathways that are responsible for initiation and progression of CRC. In colorectal cancer samples PDE6A and PDE6B genes were found to be hyper-mutated genes and all the mutations are enlisted in OASIS, a multi-omic data repository for cancer [22,42]. Expression of PDE6 isozymes has also been shown in breast cancer cell lines [18]. In the presence of somatic missense mutations, the negative regulatory effect of PDE6 on downstream subcellular signaling pathways is compromised, which could serve as an important means of acquisition of tumorigenesis in multiple experimental models of human cancer [14,30]. The role of cGMP/PKG signaling during excessive stimulation of mitogen-activated protein kinase/extracellular signal-regulated kinase (MAPK/ERK) signaling pathway has been established as central to molecular pathogenesis of approximately 40% of human melanomas. Cross talk of components of the cGMP signaling pathway and Ca^2+^ signaling attenuates MAPK/ERK proliferative signaling in melanoma cells [51]. PDE6 is crime partner of mutated p53, through which it subvert cGMP signaling pathway. PDE6 isoforms are non-canonical co-receptors and effector molecule of Wnt/Ca^2+^/NFAT signaling pathway as well [31,36]. Although experimental evidence on expression of PDE6 mutations in cancer cell lines is available [3,34] how these genomic alterations dictate normal cells to acquire a diseased phenotype is still obscure.

Among all PDEs, very little structural information about multi-domain organization of PDE6 is available as the active form of human PDE6 is challenging to express in prokaryotic or eukaryotic expression systems [45]. To gain insight into the structural implications of PDE6 mutations in cancer and retinitis pigmentosa, a computational approach was adopted. Initially three-dimensional (3D) comparative models of multi-domain human PDE6 were built based on the solved crystal structures of closely related PDEs. In addition to experimentally known mutations, saturation mutagenesis was also performed for the residues located at the dimeric interface of the A and B chains, as well as the cGMP-binding residues of Gaf-A and the catalytic domain to study their role in the stability of the quaternary structure of the protein. The current study is focused on the use of structure-driven methods to explore the possible molecular mechanism and structural effects of disease-causing mutations in PDE6. Following this, a comprehensive knowledge-based in silico mutation analysis was carried out to predict the effects and decipher the structural basis of destabilizing mutations effecting PDE6 stability and function in normal cells.

## 2 Materials & Methods

### 2.1 Homology Modelling of PDE6

To understand the molecular architecture of human PDE6, comparative modelling was performed using Modeller v 9.20 [50]. Like all other cGMP specific PDEs, both PDE6α and β chains have tandem cGMP-binding regulatory domains known as Gaf-A & Gaf-B in the N-terminal region while the catalytic domain is located at C-terminus. Amino acid sequences of PDE6α (UniProt ID: P16499) and PDE6β chains (UniProt ID: P35913) were retrieved from UniProt. Sequences of both the chains were aligned using Clustal W [58] and CLC workbench [63]. To select closely related templates for both the chains, PSI-BLAST was performed against all the known structures in the protein Databank (PDB). Modsuite (Unpublished Skwark MJ, Ochoa-Montaño B and Blundell TL), a comparative protein modelling pipeline comprised of programs such as Baton, Fugue, Joy [40] and Modeller 9.17 [50], was used to model the PDE6. Baton is used to perform structural alignment of closely related homologs of query proteins followed by Fugue [53], a distant homolog identification tool that identifies sequence-structure correlations of query proteins with known/solved homologous. Sequence to structure alignment of Fugue is annotated/represented in Joy which gives a graphical representation of local three dimensional (3D) structural features of the protein sequence in structure alignment, which makes analysis of conserved residues more tractable [40]. Based on the Fugue alignment, Modeller uses a comparative/homology modelling approach and builds structures of target proteins by identifying the conserved structural features of closely related proteins [10]. To model Gaf-A domain in α and β chains, the crystal structures of Gaf-A domain of PDE6C in complex with cGMP (PDB ID: 3DBA [38]) and of the Gaf-A domain of PDE5 (PDB ID: 2K31) were used as templates, while for the Gaf-B domain, the solved structures of the Gaf-B domain of PDE5 (PDB ID: 3LFV [61]& 3MF0 [61]) were used. For the Gaf-B domain of PDE6β, an additional template comprised of the crystal structure comprised of PDE2 Gaf-B region (PDB ID: 3IBJ) was also used. While for C-terminal catalytic domain, the crystal structure of cGMP bound to PDE5 catalytic domain (PDB ID: 1T9S [64]) and chimeric PDE5/PDE6 catalytic domain (PDB ID: 3JWR [4]) were used as templates. Later, the crystal structure of PDE2 (PDB ID:3IBJ [43]) was used as a template to borrow the structure of the central long α-helices, on which all the individually modelled domains were assembled to makeup a cGMP bound heterodimeric complex of PDE6αβ using UCSF Chimera [46]. Root means square deviations (RMSD), DOPE score [52]and GA341 values [21] of individual domain models and final heterodimeric PDE6 model were calculated. Energy minimization of initial models was performed using MMF94s force field in UCSF Chimera and quality assessments of initial and refined PDE6αβ models in complex with cGMP were performed using RAMPAGE and Molprobity [11]. Pymol [17] was used for model analysis and figure illustrations.

### 2.2 Data Collection of PDE6 Mutations

Various experimental studies have reported missense mutations of PDE6 and their implications in cancer [8,18,60] and retinitis pigmentosa [12,19,33,37,49]. To explore the influence of these mutations on the stability of the PDE6 structure, 145 missense mutations in the PDE6A gene and 114 mutations in the PDE6B gene were retrieved from catalogue of somatic mutations in cancer (COSMIC) [25]. It is the world's largest expert-curated cancer- specific catalogue of somatic mutations. We further noticed that all the PDE6 mutations reported in the literature for retinitis pigmentosa, which include 17 and 32 missense mutations in PDE6A and PDE6B respectively, are present in COSMIC as well. In addition to this, computational saturation mutagenesis was performed for interfacial residues in order to understand how mutations affect the structural stability of the PDE6αβ heterodimeric interface. Interface residues of the PDE6αβ complex were identified using Pymol. Likewise, saturation mutagenesis was also performed for cGMP-binding domains (both Gaf-A & catalytic domains) of the PDE6 heterodimer in order to understand the impact of mutations on the Gaf-A-cGMP (protein-nucleic acid) interactions. The cGMP binding residues in Gaf-A and catalytic domains were identified using Intermezzo (Ochoa-Montaño B, Blundell TL – unpublished), an in house developed program for identifying interatomic interactions.

### 2.3 In Silico Mutation Analysis of PDE6

To study the impacts of mutations on the stability of the PDE6αβ dimer, the change in free energy of the model was estimated using programs namely mutation cut off scanning matrix (mCSM) [47] and Site Directed Mutator (SDM) [42]. Mutation cut off scanning matrix (mCSM) has several modules; protein stability (mCSM), protein-protein interaction (mCSM-PPI) and Protein-DNA (mCSM-NA). Mutation cut off scanning matrix employs machine learning and graph-based signatures which defines the wild-type residue environment based on a pairwise interatomic distance matrix. The distance matrix of wild- type residues is then represented as a feature vector and changes induced by mutations are represented as pharmacophore count vector for mutant and wild type residues. Eight atomic pharmacophore features (positive, negative, hydrophobic, positive, hydrogen acceptor and donor, sulphur, aromatic and neutral) are defined in the mCSM algorithm. An estimate of the difference between wild-type feature vector and mutant pharmacophore vector is included in the signature which also includes experimental parameters i.e. pH, solvent accessibility and temperature. Final generated signature vector is than employed to train the predictive classification and regression model, which calculates the change induced by mutations in terms of Gibbs free energy (ΔΔG) of folding. Alongside this, we also used SDM [44] to predict thermodynamic changes in PDE6 wild type and mutant structures by calculating a stability score. It calculates protein stability score by estimating the probability of amino acid substitutions using environment-dependent amino acid substitution tables derived from homologous protein families. Mutant models were generated using Andante [54], a tool integrated with SDM [44]. For mutant models, Andante [54] applies a comparative modelling approach that uses χ angle conservation probabilities to select lowest energy sidechain conformations from homolog templates to ensure minimum root mean square deviation (RMSD). To improve the overall accuracy of prediction of mutations under consideration, we also used DUET [48] that integrates both mCSM and SDM. Furthermore, mCSM-PPI was used to determine the impact of mutations on the stability of the dimeric interface and mCSM-NA for Gaf-A – cGMP interaction.

### 2.4 Selection/Filtering of PDE6 Mutations in Relation to Cancer

Frequency distributions of missense mutations in COSMIC for PDE6A and PDE6B, according to their ΔΔG values, were analyzed through histograms built using R-scripts. The effects of mutations predicted by mCSM and SDM were mapped onto the PDE6αβ complex by developing attribute files in UCSF Chimera using their ΔΔG values. Predicted effects of saturation mutagenesis on the dimeric interface and cGMP-binding regions of Gaf-A and catalytic domains were also mapped onto the PDE6αβ complex. Later, those mutations within PDE6αβ that are reported as cancer causing mutations in COSMIC were selected and stability changes were predicted. Of these mutations, we focused on residues that are important in cGMP- binding, and/or catalytic activity of PDE6αβ as well as those involved in stabilization of the PDE6αβ heterodimer. Additionally, those mutations that are common in cancer and retinitis pigmentosa were also selected and analyzed.

Later, we mapped the interatomic interactions of wild type and mutant residues of a few selected mutations in the cGMP-binding pocket and dimeric interface, using Intermezzo in order to consider the structural effects in detail.

## 3 Results

### 3.1 Homology Model of PDE6

**Fig. 1:**
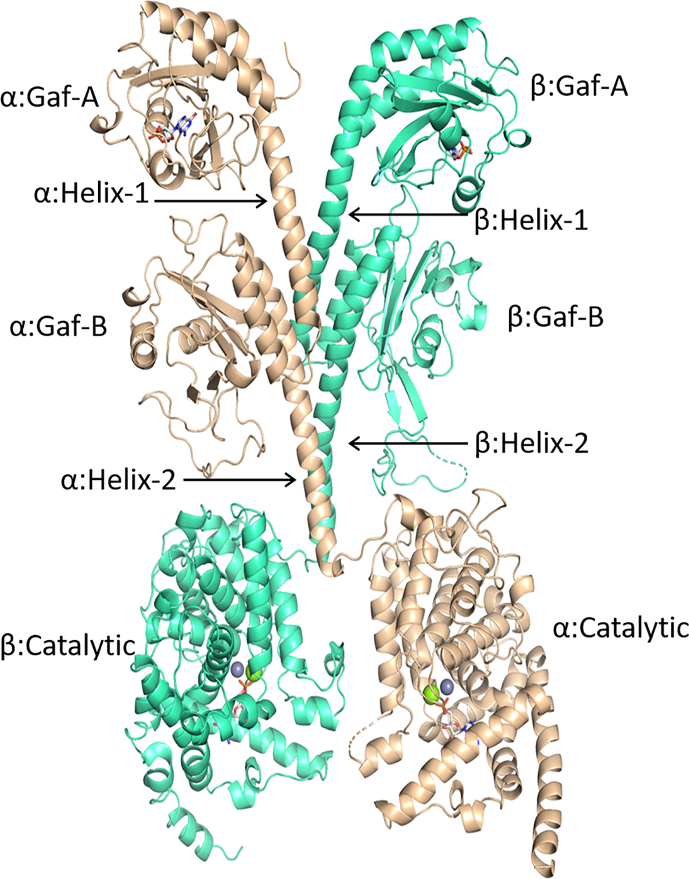
Homology model of cGMP-bound PDE6 heterodimer showing PDE6α and PDE6β chains. Each chain is comprised of Gaf-A, Gaf-B and catalytic domains connected through α-helices.

Sequence alignment of PDE6A and PDE6B revealed 71.4% sequence identity. Sequence alignment is presented in Supplementary Fig. 1. For comparative modelling, we first built models of individual domains of PDE6αβ chains encoded by PDE6A and PDE6B genes. Fugue generated sequence-to-structure alignments of individual domains of PDE6α and PDE6β chains with respect to their template structures; their predicted models are given in Supplementary Figs. 2 and 3 respectively. Root- mean-square deviations (RMSD) of all individual models were 0.2–0.6 Å with their respective templates while, Ramachandran analysis revealed 96–98% residues in favorable regions. Individual domains were then modelled in a heterodimeric structure of cGMP bound PDE6 with a dyad symmetry as shown in Fig. 1. In our predicted model, chain A (PDE6α) of PDE6 encoded by PDE6A gene includes residues from positions 53–823 while chain B (PDE6β) encoded by PDE6B gene is comprised of 68–831 amino acids. On each chain, tandem Gaf-A and Gaf-B domains are organized oriented in same direction. Catalytic domains of the two chains are rotated around the dyad axis with respect to the tandem Gaf-A & B domains (see Fig. 1). The overall molecular architecture of PDE6αβ heterodimer shows a parallel arrangement of both the chains mediated through two long juxtaposed α-helices that makeup the dimeric interface of the model. Both Gaf-A and catalytic domains in PDE6αβ have cGMP molecules bound to them and additionally two metal ions, a Mg^2+^ and a Zn^2+^ are also present in the catalytic pocket of each promoter (see Fig. 1).

The domain organization of the predicted model is consistent with the crystal structure of PDE2; however, our predicted model is in an open conformation, whereas cGMP is bound to the catalytic pocket. Unlike the crystal structure of the apo form of PDE2, the catalytic domains swing away from the dimeric interface and are not contributing to the dimer formation; rather they have an open conformation of the catalytic pocket. Quality analysis of PDE6αβ revealed 97.1% residues in the favorable regions and only 2.9% of the residues in the outlier region.

### 3.2 PDE6 Mutations Analysis

**Fig. 2:**
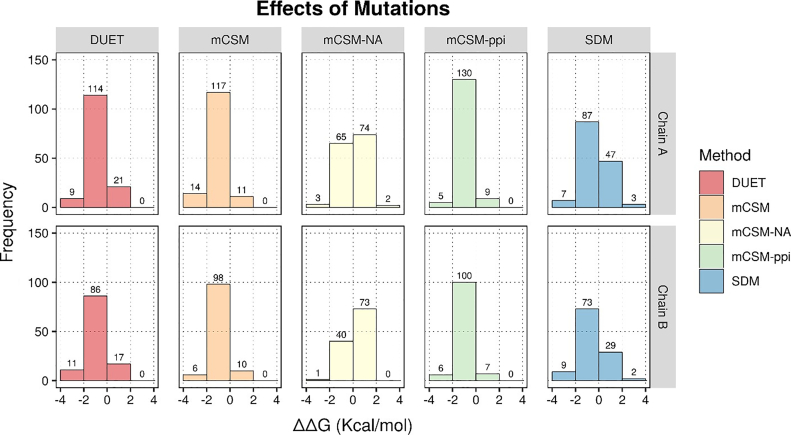
Frequency distribution graphs illustrating the distribution of experimentally known PDE6A and PDE6B mutations based on ΔΔG values predicted through DUET, mCSM-PS, mCSM-NA, mCSM-PPI and SDM. The graph bars shows stabilizing mutations (ΔΔG ≥ 0 kcal/mol), slightly damaging mutations (0 > ΔΔG ≥ −2.00 kcal/mol) and highly destabilizing mutations (ΔΔG < −2.00 kcal/mol).

**Fig. 3:**
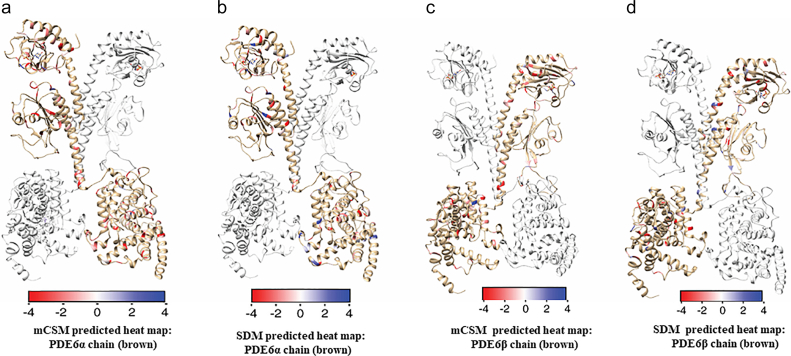
Heat maps based on mCSM and SDM predicted change in ΔΔG values of PDE6α and β chain. a) Shows mCSM while b) represents SDM predicted changes in protein stability of PDE6α chain upon COSMIC mutations. Similarly, c) shows mCSM while d) represents SDM predicted changes in protein stability of PDE6 β chain upon COSMIC mutations. Effect of mCSM and SDM predicted ΔΔG values for mutations occurring in PDE6αβ is depicted through a colored gradient scale, starting from blue indicating the average stabilizing effect (>0 kcal/mol) to highly destabilizing (<−2.00 kcal/mol) shown in red.

For chain α of the PDE6αβ heterodimer, stability changes due to 145 mutations from multiple cancers were analyzed using mCSM-PS, SDM and DUET. mCSM predicted 7.5% mutations (11/145) has stabilizing effect on PDE6 (ΔΔG > 0). While 97.5% mutations (131/145) were destabilizing (ΔΔG < 0 kcal/mol). Of the destabilizing mutations, 89% (117/132) have a mild effect on the stability of PDE6α (ΔΔG > −2.0 kcal/mol), while 12% of them are highly destabilizing mutations (ΔΔG < −2.00 kcal/mol) (Fig. 2). mCSM predicted stability changes were mapped onto the structure and are shown in Fig. 3a. Stability changes (ΔΔG) predicted using mCSM -PS and SDM showed that the majority of the mapped missense mutations are mildly affecting the stability and few positions having high destabilizing effects. Using SDM, 60% of the mutations (87/145) were predicted to confer mild changes in stability while approximately 5% (7/145) were shown to be highly destabilizing (Fig. 2). SDM predicted energy changes were mapped onto the PDE6α chain and are shown in Fig. 3b. DUET predicted 78.6% (114/145) as mildly destabilizing mutations while approximately 6% (9/145) are predicted as highly destabilizing (ΔΔG < 0 kcal/mol) (Fig. 2).

To predict the impact of mutations on the protein- protein interfaces, mCSM-PPI program was used. mCSM-PPI reveals only 3.7% (5) mutations as highly destabilizing of interdomain, interchain or multimeric signaling partner interactions. 89.6% of mutations (130/145) in the α-chain were predicted to have mild effects on the protein- protein interfaces as their ΔΔG values range from −0.044 to −1.804 kcal/mol. To understand the impact of these missense mutations on binding of cGMP at Gaf-A domain, mCSM-NA was applied. 45% (65/145) were predicted to destabilize cGMP binding, while 4 mutations in the binding pocket were shown as highly destabilizing which may disrupt cGMP binding with Gaf-A domain (Fig. 2).

For the β chain of the PDE6αβ heterodimer, 114 missense mutations reported from multiple cancers were analyzed in the current study. mCSM predicted approximately 9% of the mutations (10/114) to be stabilizing PDE6, while 91% of mutations (104/114) were predicted to be destabilizing. Out of these 114 destabilizing mutations, approximately 94% (98/104) are mildly destabilizing (ΔΔG > −2.0 kcal/mol) while around 6% (6/104) were predicted as highly destabilizing (ΔΔG < −2.00 kcal/mol) (Fig. 2). Stability changes predicted for 145 mutations were mapped onto the structure (Fig. 3c). Using SDM, 64% (73/114) of the mutations were found to affect protein stability and approximately 8% were predicted to highly destabilize PDE6β (9/114). DUET revealed 75% mutations as slightly destabilizing and approximately 10% as highly destabilizing (Fig. 2). SDM predicted stability changes were mapped on the PDE6β structure as shown in Fig. 3d. Stability changes due to mutations in PDE6β at the subunit interface with PDE6α (predicted using mCSM-PPI) indicated that 6.1% (7) are stabilizing mutations, whereas 5% (6/114) are highly destabilizing which may impact the heterodimer formation. The remaining 88% of the mutations in PDE6β chain were predicted to have mild destabilizing effects on protein- protein interfaces as their ΔΔG values lie in range of −0.4 to −1.9 kcal/mol. Mutations that affect the cGMP binding were predicted using mCSM-NA and the results show 35% of the mutations (40/114) to be slightly destabilizing cGMP binding whereas only one mutation was found to be highly destabilizing and probably disrupt cGMP binding in the catalytic pocket/Gaf-A domain (Fig. 2). Furthermore, mutation analysis of 17 retinitis-pigmentosa-specific missense mutations in PDE6A showed that 9 mutations were also present in COSMIC databases, out of which 8 were found to be slightly destabilizing in terms of their ΔΔG values (Supplementary table 1). While in PDE6β, only four missense mutations were found common among cancer and retinitis pigmentosa. Mutation analysis through aforementioned tools revealed that three of them were slightly destabilizing (see Supplementary table 1), while the missense mutation of the highly conserved catalytic residue (D600N) reported, in both PDE6α and PDE6β chains, is significantly destabilizing the binding of metal ions in the cGMP-binding pocket of the catalytic domain.

#### 3.2.1 Experimentally Known PDE6 Mutations for Cancer

##### 3.2.1.1 PDE6α Gaf-A Domain Missense Mutations; F162L, V141M, R93L

**Fig. 4:**
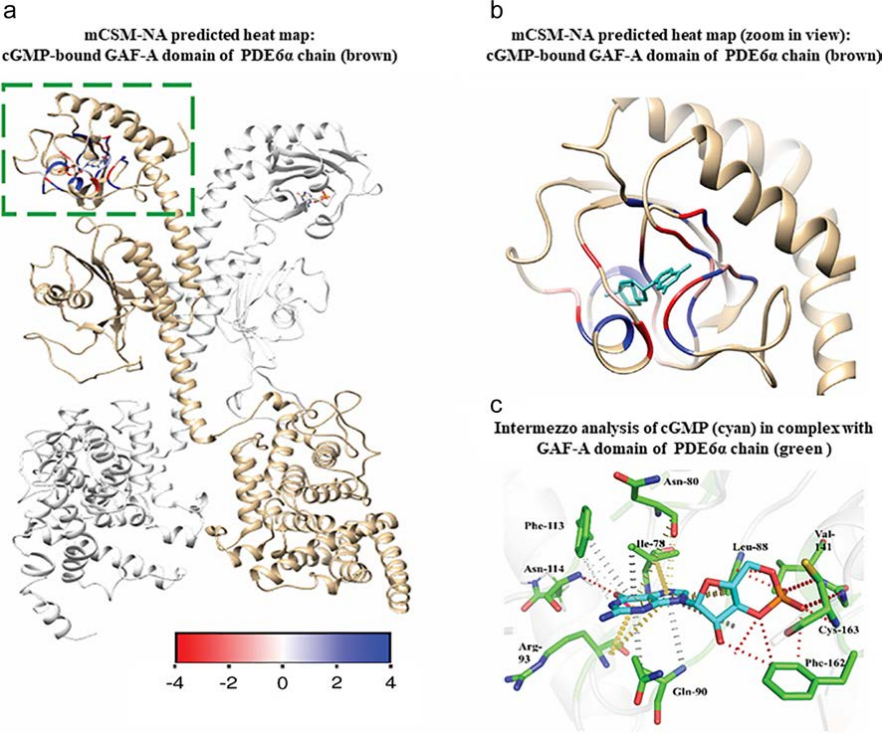
a) Heat map showing the average predicted changes upon mutation in cGMP-binding residues of PDE6α Gaf-A domain using mCSM-NA (b). zoom in view of cGMP bound Gaf-A domain and predicted effect of mutations in cGMP interacting residues of Gaf-A domain is shown through a colored gradient scale that starts from blue indicating the average stabilizing effect (>0 kcal/mol), slightly destabilizing (≥−2.00 kcal/mol) (white) and highly destabilizing (<−2.00 kcal/mol) (highlighted in red). c) depicts Intermezzo analysis of cGMP binding with the wild type residues of Gaf-A domain of PDE6 through non-covalent interactions. Red dotted lines indicate hydrogen bonds as well as van der wal interactions of the cGMP molecule with Leu-88, Asn-114, Val-141, Phe-162 and Cys-163. Hydrophobic interactions involving ring-ring and ring-atom interactions between cGMP molecule and Phe-162, Ile-78, Gln-90 are shown in grey color while inter-residue interactions of Ile-78, Asn-87 and Arg-93 with the ring atoms of the cGMP molecule are shown in light yellow color.

Impact of mutations on the cGMP binding was predicted using mCSM-NA and the stability changes were mapped on the Gaf-A domain (Fig. 4a and b). In the Gaf-A domain of PDE6α, Lys-76, Ile-78, Leu-88, Asn-80, Gln-90, Arg-93, Phe-113, Asn-114, Val-141, Cys-163 and Phe-162 interact with cGMP (Fig. 4c). Backbone atoms of Ile-78, Asn-114, Val-141 and sidechain atoms of Leu-88, Ile-140, Phe-162 form hydrogen bond and van-der-Waals interactions with the cGMP molecule as shown in Fig. 4c. Ring atoms of Phe-113 form π-π stacking interaction with the purine ring of cGMP. Mutations in the residues that interact with cGMP resulted in the loss of native binding interactions which might affect the cGMP binding to Gaf-A domain.

**Fig. 5:**
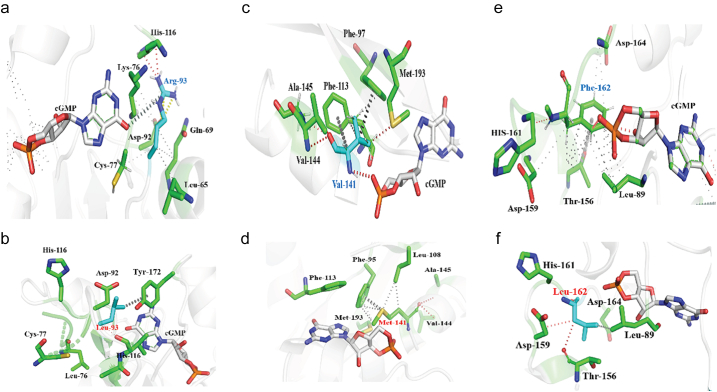
a) illustrates interactions between Arg-93 (R93 in cyan color) with the cGMP molecule (white) as well as with the neighboring residues (green color) of the wild-type PDE6αβ. Hydrogen bonds (red dotted lines), proximal hydrophobic interactions (small grey dotted lines), ionic interactions (yellow dashed lines) and carbon-π interactions (white dotted lines). b) Depicts carbon-π interaction (grey dotted line) between mutant Leu-93(labelled in red color) and Tyr-172 while loss of interactions with neighboring environment residues and cGMP molecule can also be observed. c) Depicts interactions of Val-141(cyan) with cGMP molecule (white) and neighboring residues (green) in the environment of the wild-type PDE6αβ complex. Hydrogen bonds and van der wal interactions are shown by red lines; hydrophobic interactions (small grey dotted lines) and carbon-π interactions of Val-141 with the ring atoms of Phe-97 and Phe-113 are shown in dark grey dotted lines. d) Represents mutant Met-141 (labelled in red) binding with nearby residues (green) of the environment through various non-covalent interactions; hydrogen bonds (red dotted lines), hydrophobic interactions (light grey dotted lines) and methionine-π interaction (dark grey dotted lines) with ring atoms of Phe-97. Loss of interaction between cGMP molecule and mutant V141 M is also shown. e) Illustrates non-covalent interactions of wild type F162 (labelled in blue with cGMP (white) and nearby residues (green). Hydrogen bonds are shown in red dotted lines, proximal hydrophobic interaction in small grey dotted lines while inter-residue donor-π interactions are highlighted in dark grey dotted lines. f) represents binding of F162 L mutant (labelled in red) with its three nearby residues (leu-89, Asp-159, Thr-156) through weak hydrogen bonds (red dotted lines) and hydrophobic interactions (small grey dotted lines). Loss of binding with cGMP molecule is also evident mutant complexes (b,d,f).

Three mutations in the Gaf-A domain that are earlier reported in COSMIC and are known to highly destabilize cGMP-Gaf-A interactions are discussed below:

R93L: In wild type PDE6A, sidechain atoms of Arg-93 interacts with the cGMP molecule by making a non-bonded edge-to-tilted (ET) interaction. Arg-93 also forms hydrogen bonds with His-116 and an ionic interaction with Asp-92. Proximal hydrophobic interactions were also observed with Leu-65, Cys-77 and Lys-76. These interactions of Arg-93 with the neighboring residues contribute to the shape and folding of the cGMP-binding pocket in Gaf-A. For R93L mutation, mCSM-PS, SDM and DUET predicted ΔΔG values as −1.211 kcal/mol, −1.01 kcal/mol and −1.297 kcal/mol respectively, which shows that this mutation is mildly destabilizing. However, predictions by mCSM-NA revealed that this mutation is highly destabilizing to cGMP binding (ΔΔG = −2.22 kcal/mol) (See Table 1). Further, analysis of interatomic interactions using Intermezzo revealed loss of vital interactions with cGMP molecule as well as loss of native interactions with the surrounding residue environment i.e. Asp-92, His-116, Leu-76, Cys-77 (Fig. 5b).

**Table 1:** List of experimentally known mutations for Cancer in PDE6A Gene encoding Gaf-A and Catalytic Domain of PDE6α chain (predicted ΔΔG in kcal/mol).

Mutations	Gene	mCSM	SDM2	DUET	mCSM-PPI	mCSM-NA
PDE6A Gaf-A						
F162 L	PDE6A	−1.381	−1.95	−1.492	−1.027	−0.549
V141 M	PDE6A	−0.951	−0.98	−0.61	−1.576	−4.978
R93L	PDE6A	−1.211	−1.01	−1.297	−1.358	−2.222

PDE6A catalytic domain						
D600N	PDE6A	−1.011	−0.934	−1.554	−1.287	−3.088
F742 L	PDE6A	−1.751	−0.872	−0.706	−1.512	−1.702
F776 L	PDE6A	−1.504	−0.72	−0.706	−1.281	−1.702

V141 M: Valine at position 141 binds to the cGMP molecule and neighboring backbone and sidechain atoms of Val-144 and Ala-145 respectively through van der Waals interactions. Sulphur group of Met-193 interacts with Val-141 through weak van der Waals interaction. Within its surrounding residue environment, Val-141 also forms a carbon-π interaction with the ring atoms of Phe-97 and Phe-113 (Fig. 5c). In the mutant form, Val-141 is substituted with a methionine, which induces loss of binding with the cGMP molecule in addition to the change in the binding pattern with the surrounding residues. Mutation analysis using mCSM, SDM and DUET and mCSM-PPI predicted it as a mildly destabilizing mutation in terms of protein stability whereas mCSM-NA indicated that mutation would highly destabilize cGMP binding (ΔΔG = −4.978 kcal/mol). V141M brings about a change in the binding pocket resulting in the loss of its carbon-π interaction with Phe-113 and van der Waals interaction with the Met-193 as wild type, while it retains van der Waals interactions with the backbone atoms of Val-144 as well as with side chain atoms of Ala-145. The mutant also forms methionine-π interaction with ring atoms of Phe-97 and hydrophobic interaction with Leu-108. Interaction of the mutant residue with the surrounding residue environment other than the native ones in the wildtype, might affect the binding pocket conformation and impact the cGMP binding (Fig. 5d).

F162L: F162L, another missense mutation within the binding pocket of the Gaf-A domain, was predicted to have a mild destabilizing effect (predicted by mCSM, DUET and SDM to be −1.381 kcal/mol, −1.95 kcal/mol, −1.492 kcal/mol respectively). This mutation mildly destabilizes subunit interface and the cGMP binding (see Table 1). In wild-type, F162 ring forms three weak hydrogen bonds (van der Waals) with the cGMP molecule. The side chain oxygen atom of Thr-156 forms a donor-π interaction with the ring atoms of F612 and it also shares a dense network of proximal hydrophobic interactions with the sidechain atoms of Leu-89, Asp-159, His-161 and Asp-164 (Fig. 5e). Intermezzo analysis revealed change in the cGMP binding pocket which resulted in the loss of weak hydrogen bonds with cGMP as well as hydrophobic interactions with His-161 and Asp-164. Additionally, the mutant forms weak hydrogen bonds with Asp-159 and Thr-156 as well as hydrophobic interaction with Thr-156 (Fig. 5f).

#### 3.2.2 PDE6A Catalytic Domain

**Fig. 6:**
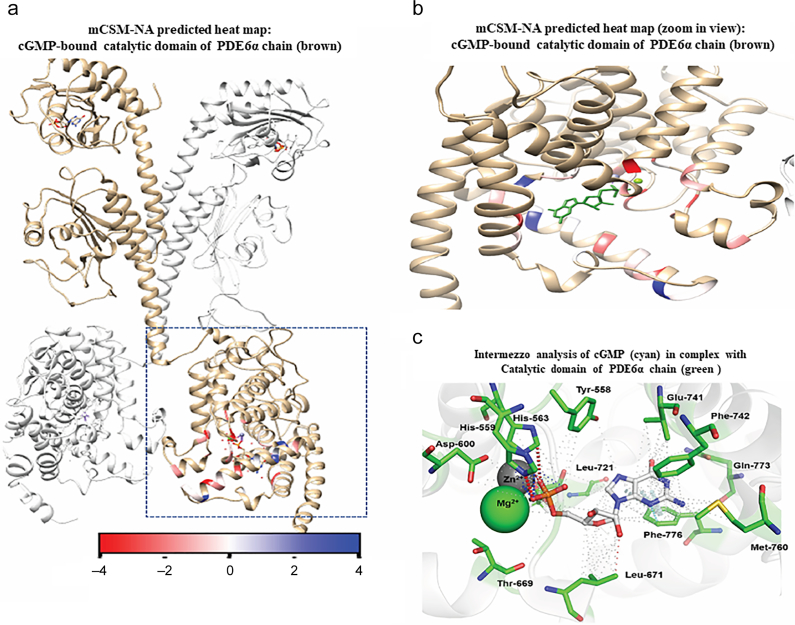
a) Heat map based on mCSM-NA predicted ΔΔG values showing the average stability changes upon mutation in cGMP binding residues of PDE6α catalytic domain. (b). Zoom in view illustrating the average effect of mutations in critical catalytic residues that binds cGMP molecule. Colored gradient scale shows the mutation effects; stabilizing (>0.00 kcal/mol) (blue), slightly destabilizing (≥−2.0 kcal/mol) (white) and highly destabilizing (<−2.00 kcal/mol) (highlighted in red) c) Intermezzo analysis depicts interatomic interactions between cGMP molecules and wild type residues of catalytic domain of PDE6 through non-covalent interactions. Red dotted lines indicate ionic bonds between His-563, His-559 and Zn^2+^ ion. Metal interactions involving Zn^2+^ ion and cGMP molecule are shown in blue color. Weak hydrogen bonds are shown in small red dotted lines. π-π stacking interaction and aromatic interaction of Phe-776 with the cGMP molecule within the catalytic pocket are shown in white and cyan colored dotted lines respectively. Other important catalytic residues i.e. Tyr-558, Asp-600, Thr-669, Leu-671, Glu-741, Phe-742, Met-760, Gln-773 are shown to have undefined interaction with the cGMP molecule depicted in grey small dotted lines.

In addition to the non-catalytic residues, the effect of missense mutations on catalytic residues were also analyzed in the current study. In PDE6α and PDE6β, catalytic residues within the active site are conserved. Within the catalytic pocket, two metal ions (Mg^2+^, Zn^2+^) and Tyr-558, His-559, His563, Asp-600, Thr-669, Leu-671, Glu-741, Phe-742, Met-760, Gln-773 and Phe-776 were observed to have a dense network of interactions with the cGMP molecule (Fig. 6c). Effects of mutations in catalytic residues in terms of affinity changes of cGMP molecules with catalytic domain are predicted through mCSM-NA and are mapped on catalytic pocket of PDE6α (Fig. 6a & b). Sidechain atoms of His-563, His-559 formed a network of ionic bonds with Zn^2+^ ion. Interactions of Mg^2+^ and Zn^+2^ ions with cGMP molecule can also be seen in Fig. 6c. Ring-to-edge interactions of Asp-720 and π-π stacking interaction of Phe-776 may contribute to the orientation of cGMP molecule in the catalytic pocket. Other important catalytic residues i.e. Tyr-558, Asp-600, Thr-669, Leu-671, Glu-741, Phe-742, Met-760, Gln-773 were observed to have proximal interactions with the cGMP molecule. All the residues given in Fig. 6b are conserved catalytic residues of the phosphodiesterase family [6,65]. mCSM-NA analysis of saturated mutations in the catalytic pocket revealed highly destabilizing effects for three mutations which are discussed below. These mutations are also reported in the COSMIC database. Of these three highly destabilizing mutations, one is D600N which is reported to be a missense mutation in the catalytic pocket of PDE6α and PDE6β in retinitis pigmentosa as well as in the COSMIC database.

**Fig. 7:**
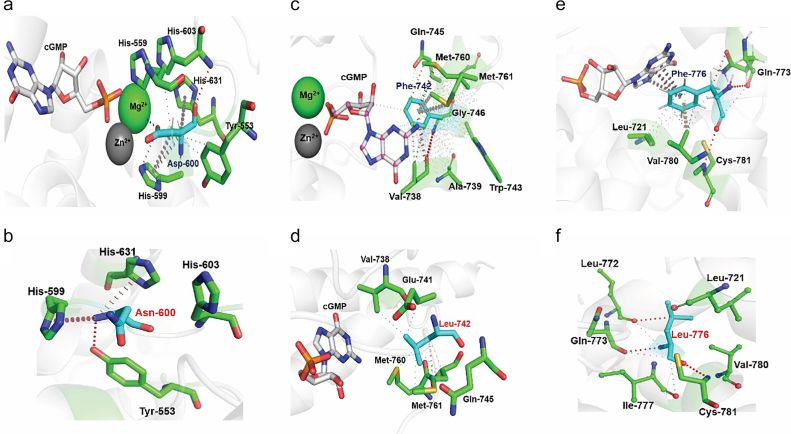
Interatomic interaction analysis of wild-type and mutant PDE6 catalytic residues a) depicts Asp-600 (cyan colored) bound to cGMP (white), Mg^2+^ (green sphere) and nearby residues (green) within the catalytic pocket. Ionic interactions (blue), hydrogen bonds and *van der* Waals (red color), hydrophobic interaction (grey small dotted lines) and carbon-π interactions (dark grey dotted lines). b) Illustrates interaction network of missense mutation Asn-600 (labelled in red) with cGMP (white), Mg^2+^ (green sphere) and surrounding catalytic residues (green). Hydrogen bonds are shown in red dotted lines whereas donor-π interaction with wheat colored dotted lines. c) Shows dense network of hydrophobic and ring interactions between Phe-742 (cyan), cGMP (white) and surrounding catalytic pocket's residues (green). Hydrogen bonds are depicted in red dotted lines, atom-ring interactions including methionine -π interactions and carbon-π interactions are shown in dark grey dotted lines. Proximal hydrophobic interactions (small grey-dotted lines) and undefined interactions are shown as dense network of small dots. d) Illustrates dense proximal hydrophobic interaction network (small grey dotted lines) of missense mutation F742 L (Leu-742 labelled with red) with neighboring residues (green). Loss of interaction between cGMP (white) and Leu-742 is also depicted in graphical depiction. e) graphical illustration of Intermezzo analysis of F776 (cyan) with cGMP (white) and nearby surrounding residues (green) of catalytic domain of PDE6. Hydrogen bonds are shown in red dotted lines, hydrophobic interactions are shown in small dotted lines, ring-ring interactions between F776 and cGMP are shown in grey dotted lines while carbon-π interactions of F776 with neighboring residues are shown in wheat color. f) Illustrating hydrogen bond (red dotted lines) and carbonyl interaction (magenta color) of missense mutation Leu-776 (F776 L labelled in red) with neighboring residues (green). Loss of binding between F776 L and cGMP was also observed in intermezzo analysis.

D600N: In the catalytic pocket of PDE6α and PDE6β, Asp-600 is highly conserved residue that binds to Mg^2+^ and Zn^2+^, ion which in turns interacts cGMP molecule. In the surrounding residue environment, Asp-600 forms *van der* Waals interactions with His-603 and sidechain atoms of His-559 and Tyr-553 form proximal hydrophobic bonds with Asp-600. Backbone atoms of Asp-600 are also making carbon-π interaction with the His-631 and His-599 as depicted in Fig. 7a. Four histidine residues, i.e. His-559, His-599, His-603, His-631, bind to the Zn^2+^ ion which in turn regulate the catalytic activity of PDE6. Missense mutation D600N in PDE6α as well as in PDE6β has been reported both for cancer and retinitis pigmentosa of photoreceptor cells. Analysis of D600N using mCSM-PS, SDM, DUET and mCSM-PPI predicts it as a mildly destabilizing mutation (see Table 1). However, mCSM-NA predicted it as a highly destabilizing mutation (ΔΔG value <−2.00 kcal/mol) (Table 1). Intermezzo analysis showed D600N exhibited loss of ionic bonding between Mg^2+^ and mutant residues. With the residues in the surrounding environment, D600N mutant shows donor-π interaction with His-559 and His-631 while loses its native hydrophobic interaction with other two histidine residues. In D600N, side chain atoms of Tyr-553 form hydrogen bond with Asn-600 as shown in Fig. 7b.

F742L: Ring atoms of F742 forms one edge-to-edge and one atom-ring interaction with the cGMP molecule. While with the neighboring residues, backbone atoms of F742 forms van der Waals interaction with the backbone and sidechain atoms of Val-738 and Ala-739. Additionally, side chains of Val-738, Gln-745 and Met-761 interact with Phe-742 through hydrophobic interactions. In addition to this, side chain atoms of Met-760 make methionine-π and carbon-π interaction with the ring atoms of Phe-742 as shown in Fig. 7c. Mutation analysis of F742 L showed it a mildly destabilizing mutation when analyzed through mCSM, SDM, DUET, mCSM-PPI and mCSM-NA as all of them predicted ΔΔG score < 0 (see Table 1). Intermezzo analysis of F742L revealed loss of hydrophobic interaction between ring atoms of F742 and cGMP. Backbone atoms of Leu-742 form a dense network of hydrophobic interactions with the sidechains of surrounding residues i.e. Val-738, Glu-741, Met-760, Met761 and Gln-745 as shown in Fig. 7d.

F776L: In wild type PDE6α, ring atoms of Phe-776 is forming ring-ring interaction with the purine ring of cGMP. In addition to this, sidechain atom of the Phe-776 is also making a hydrogen bond with the edge of cGMP ring structure. Further, carbon-π interaction is observed between the sidechain atoms of cGMP molecule and Val-780 and Gln-773 is interacting with the backbone atom of F776 through two hydrogen bonds as shown in Fig. 7e. Missense mutation F776L show mild destabilizing effect (Table 1) as predicted by mCSM, SDM, DUET, mCSM-PPI and mCSM-NA analysis. Intermezzo analysis of F776L exhibited loss of ring - ring and hydrogen bonding with cGMP molecule. With the surrounding environment Leu-776 interacts with Leu-772, Gln-773 and Cys-781 through hydrogen bonds while backbone atoms of Leu-776 form carbonyl interaction with the sidechain atoms of Ile-777 and Gln-773 (shown in Fig. 7f).

### 3.3 Mutation Affecting PDE6αβ Dimer Interface

The heterodimeric interface of PDE6 αβ complex is stabilized by two long juxtaposed α-helices, which acts as a backbone in the structure. The central helices were built based on experimentally known template structure of PDE2. To understand the impact of mutations on the subunit interfaces, we carried out computational saturation mutagenesis of interface residues. mCSM-PPI analysis revealed 94% mutations as mildly destabilizing while 26% were predicted as highly destabilizing mutations in interfacial residues of the PDE6α chain. Whereas approximately 97% mutations of interfacial residues are mildly destabilizing, 28% mutations are highly destabilizing mutations in the PDE6β chain as shown in Supplementary Fig. 4a. The predicted effects of the mutations were mapped onto the central helical regions of PDE6αβ dimer (Supplementary Fig. 4b and c). The regions shown in red color represent the dimer interface residues, which upon mutation might disrupt the intertwined helical organization and ultimately result in loss of PDE6αβ structural stability.

At the heterodimeric interface of the PDE6αβ complex two residues (Phe-249 and Phe-426) were observed to form a network of hydrophobic, inter-residue, ring-ring and atom-ring interactions with Phe-424. Mutations in these residues are discussed below in detail.

**Fig. 8:**
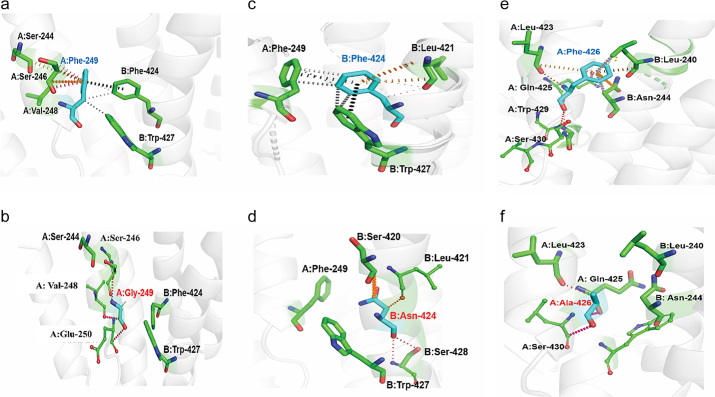
a) Graphical description of wild type Phe-249 (cyan) present at the dimeric interface of PDE6A chain that is found to establish interaction with interfacial residues of PDE6B (Phe-424 and Trp-427). Hydrogen bonds are shown in red dotted lines, hydrophobic interactions are highlighted in grey dotted lines. Ring-ring (π-π stacking) interactions are highlighted in black while inter residue interactions are shown in orange color. b) shows mutant Gly-249 (labelled with red) present at the dimeric interface of PDE6A, which establishes hydrogen bond (red dotted lines) and carbonyl interactions (magenta dotted lines) with neighboring residues that are present within its chain while disrupting strong interactions at protein-protein interface residues as predicted by mCSM-PPI c) shows residue Phe-424 (cyan) performing a dense network of hydrophobic (grey dotted lines), ring-ring (black dotted lines), atom-ring interactions (magenta dotted lines) and inter residue interactions (orange) with neighboring residues of its PDE6A chain as well as with interfacial residues of PDE6B chain. d) Mutation to Asparagine (Asn-424) in PDE6B is predicted to be highly destabilizing, disrupting dimer interaction with Phe-249 present at PDE6A as predicted by mCSM-PPI. While Asn-424 retains carbonyl interactions (magenta dotted lines), hydrogen bonds and van der wal interactions (red dotted lines) and inter-residue interactions (orange) with neighboring residues that are present within its chain. e) represents PDE6A chain residue Phe-426, present at dimer interface performing atom-ring interactions (grey dotted lines) and inter residue interactions (orange) with interfacial residues of PDE6B while it also establishes hydrogen bonds (red dotted lines) with other residues of same chain. f) Mutation to Ala-426 in PDE6A is forming carbonyl (magenta) and hydrogen bonds (red dotted lines) with the nearby residues of its chain while losing affinity with PDE6B interfacial residues as predicted by mCSM-PPI.

F249G: In PDE6α chain, ring atoms of Phe-249 forms ring-to-edge interactions with the Phe-424 in PDE6β chain. Helices of PDE6α and β chains are also stabilized by the hydrophobic interaction between the sidechain ring atoms of Phe-249 and Trp-427 respectively. In the surrounding environment Phe-249 forms inter residue atom-ring interaction with Ser-244 and Ser-246 (Fig. 8a) which might help in the spatial orientation of Phe-249 as an interfacial residue. The F249G mutation was predicted as mildly destabilizing mutation by mCSM- PPI (ΔΔG = −1.288 kcal/mol) (Table 2), which results in the loss of dimeric interface interaction between PDE6β chain residues (Phe-424 and Trp-427) (see Fig. 8b).

**Table 2:** Effect Of Mutations on Dimerization of PDE6αβ Complex Predicted Through mCSM-PPI (predicted ΔΔG in kcal/mol).

Mutation	GENE	mCSM-PPI
F426A	PDE6A	−2.548
F249G	PDE6A	−1.288
F424 N	PDE6B	−1.591

F424N: In PDE6β chain, Phe-424 stabilizes the heterodimer complex through a mesh of hydrophobic and aromatic interactions involving the ring atoms of Phe-424(PDE6β) and Phe-249 (PDE6α). In the environment, Phe-424 makes ring-ring (π-π) stacking interaction with ring atoms of Trp-427, it is also stabilized by the ring -edge inter-residue interaction with Leu-421 (see Fig. 8c). F424N was predicted as a mildly destabilizing mutation with mCSM-PPI predicted ΔΔG of −1.591 kcal/mol as shown in Table 2. Intermezzo analysis indicated a loss of inter-chain interaction with Phe-249 (PDE6α), Asn-424 shows van der Waals interactions with Leu-421. It also has inter-residue ring and pi-group interaction with Ser-420 (see Fig. 8d).

F426A: At the dimeric interface, F426 in the PDE6α chain forms a ring-edge hydrophobic interaction with Leu-240 and it also makes a hydrogen bond with the Asn-244 of PDE6β chain, and an inter-residue interaction with the backbone atoms of Leu-240 and Asn-244. In the PDE6α chain it interacts with surrounding residues (Ser-430 and Leu-423), as shown in Fig. 8e. The F426A mutation is predicted to highly destabilize the dimeric interface interaction (ΔΔG = −2.548 kcal/mol), in which the alanine substitution results in the loss of interactions with Leu-240 and Asn-44 of the PDE6β chain. Ala-426 forms interactions with the sidechain atoms of neighboring residues (Leu-423 and Ser-430) as shown in Fig. 8f.

## 4 Discussion and Conclusion

cGMP-specific phosphodiesterases regulate a myriad of cellular signaling processes through critical regulation of the cGMP messenger molecule and any disruption/dysregulation in the signaling pathways may lead to downstream pathophysiological impacts [2].

Evidence of association of PDE6 with cancer progression and vision loss, will be strengthened if we are able to estimate the structural and thermodynamics effects of mutations in cGMP signaling proteins. To decipher the impacts of mutations on protein stability, structural information of PDE6 is necessary to piece together the molecular details of its activation and deactivation. Although crystal structures of individual domains of different isoforms of PDE6 have been determined and a topological architecture depicting PDE6 domain's assembly is available at a very low resolution, a complete multimeric structure is not available for PDE6 for rod cells in human retina [29,57]. It is challenging to obtain an adequate purified form of PDE6 protein in an active form via recombinant (eukaryotic/prokaryotic) expression systems to solve the multimeric assembly of the structure [15]. To predict the structure of multiprotein assembles is a challenging task for structural bioinformatics. In a protein family, residues present within a domain share a strong interaction network of almost conserved residues but when multiple domain are involved, it becomes difficult to predict their spatial assembly as a fewer residues are involved in domain-domain interaction and conservation among these residues is not extensive. In the PDE6 heterodimer structure, a divide and conquer rule was followed in which individual domains were modelled by taking the advantage of the high sequence identity with their homologous. Individual domain models with high prediction accuracy were then assembled on the long interconnecting helices (substructures) borrowed from the crystal structure of PDE2 which shares less similarity with the PDE6 at the domain level. Thus, PDE6 domains were built using PDE5 and PDE6C domains and information of juxtaposed helices and domain assembly was inferred from the solved structure of PDE2. Unlike PDE2, PDE6αβ is a heterodimer, which has about 70% in sequence identity between the chains while the structure is in open conformation with cGMP bound within the conserved cGMP binding and catalytic pockets. In the past few years, sequencing of the cancer genome has yielded a rich amount of mutation data for PDE6 α and β chains. To define the mechanistic impacts of PDE6 mutations in cancer, we have described here an *insilico* workflow for the analysis of relative changes in protein structure and stability due to missense mutations. We have discussed the effects of mutation with respect to change in protein stability and its interaction pattern with the cGMP molecule and its surrounding residue environment in order to unravel the link between the mutation and disease phenotype. We identified mutations that are highly destabilizing with respect to cGMP binding and/or metal ion binding and in addition, have disruptive impact on the dimeric interface.

We also observed that missense mutations in PDE6 that are reported in COSMIC for cancer are also reported for retinitis pigmentosa in various experimental studies [28,33]. Earlier it was believed the PDE6 is only expressed in photoreceptor cells but now it is evident from various studies that retina -specific PDE6 is also expressed in the perinuclear region of endothelial cells and has a significant role in compartmentalized cGMP signaling pathways of endothelium [8]. The presence of PDE6 isozymes in breast cancer cell lines [18] suggest that in cancer the negative regulatory mechanism of PDE6, which maintains the concentration of cGMP, is no longer active and this affects the downstream subcellular signaling pathways [14].

The high frequency of PDE6 mutations in skin cancer suggest a possible crosstalk of cGMP signaling pathway and mitogen-activated protein kinase/extracellular signal-regulated kinase (MAPK/ERK) signaling pathway responsible for approximately 40% of human melanomas. Cross talk of components of the cGMP signaling pathway and Ca^2+^ signaling attenuates MAPK/ERK proliferative signaling in melanoma cells suggest that any change/mutation and disruption in cGMP signaling proteins will be negatively affecting MAPK/ERK proliferative signaling in melanoma cells [51].

Deciphering the specific role of mutations as etiological factors for various diseases relies on their ability to induce structural alterations in proteins to an extent that they influence pathophysiological outcomes. Unlike infectious diseases where genomic mutations in pathogens are most likely attributed to either resistant or virulent strains, mutations in human genes that drive various cancers and influence different cell signaling mechanisms, needs comprehensive understanding at the subcellular and structural levels. The current study is primarily focused on understanding the structural organization of the PDE6αβ complex and the impact of mutations on cGMP binding, catalytic activity and heterodimer assembly. The objective of this study was to understand the possible structural effects of experimentally-known missense mutations on the recognition of the activator molecule (cGMP) within the allosteric and catalytic binding pockets. We also evaluated and discussed the structural variations and change in binding affinity of the PDE6 dimer, because missense mutations at the dimer interface residues may affect structure and function of PDE6. In the absence of an experimentally solved structure for the full chain of a multidomain protein like PDE6, it is extremely challenging to model a plausible conformer with accurate orientation of the domains. Although the template identities are low and specific to individual domains, we were able to model the full chain heterodimeric complex of PDE6 and reasonably retain the multi-domain architecture of eukaryotic phosphodiesterases.

In conclusion, we modelled the structure of human PDE6αβ complex and used a set of state-of-the-art computational tools to understand the effects of mutations on thermodynamic stability, stability of the dimer interface, interactions with cGMP and catalytic activity. Structural changes due to mutations that alter binding patterns of cGMP in catalytic and Gaf-A domain were analyzed using experimentally known and a computationally saturated set of mutations. Further, the saturation mutagenesis of residues in the heterodimeric interface was also analyzed to provide prospective information about the possible effects of mutations if they occur at the dimer's interfaces. While most of the observations are computationally simulated, they provide a basis for prioritization of future experimental approaches to establish the structural link between mutations in PDE6 and phenotypic outcomes like cancer and retinitis pigmentosa.

## Conflict of Interest

All authors have no conflict of interest related to this study.

## Funding

The authors would also like to thank the International Research Support Initiative Program (IRSIP) of the Higher Education Commission (HEC), Pakistan, Gates Foundation, the Cystic Fibrosis Trust and American Leprosy Missions for the support of this research.

## Author Contributions

T.LB, A.R.S, S.C·V and A.M conceived and designed this study; A.M, S.C·V, A.F·S. and P.H.M.T performed protein structure modelling. A.M, S.C·V, and R.R.K collected, analyzed the data. A.M, A.F.S and P.H.M.T prepared the figures presented in the manuscript. A.M wrote the manuscript; T.L.B, A.R.S. and S.C·V improved and revised the manuscript. T.L.B provided overall support and guidance for the computational analysis. All the authors approved the final version.

## Appendix A Supplementary data

Supplementary material 1: Fig. 1 Sequence alignment of Human PDE6α and PDE6β chains using CLC workbench. Identical residues among both the chains are shown in black color while consensus residues, conservation bars plots (0-100) and sequence logos are also given at the bottom to highlight conservation in both the chain’s amino acid sequence. Image 1

Supplementary material 2 Image 2
