# Supplementary material for: The Molecular Organization of Human cGMP Specific Phosphodiesterase 6 (PDE6): Structural Implications of Somatic Mutations in Cancer and Retinitis Pigmentosa
Source: Comput Struct Biotechnol J. 2019 Mar 6;17:378–89. doi: 10.1016/j.csbj.2019.03.004 (PMC6434069; doi:10.1016/j.csbj.2019.03.004)
Supplement: Supplementary file 1 — Supplementary material 1: Fig. 1 Sequence alignment of Human PDE6α and PDE6β chains using CLC workbench. Identical residues among both the chains are shown in black color while consensus residues, conservation bars plots (0-100) and sequence logos are also given at the bottom to highlight conservation in both the chain’s amino acid sequence. [file mmc1.pdf]

```
PDE6A_Human  MGEVTAEEVEKFLDSNIGFAKQYYNLHYRAKLISDLLGAK---EAAVDFSN  48
PDE6B_HUMAN  MS-LSEEQARSFLDQNPDFARQYFG----KKLSPENVAAACEDGCPPDCDS  46
Consensus    M*E***E****FLD*N**FA*QY**LHYR*KL******A*CED****D***
Conservation (100% / 0%)
Sequence logo (4.3bits / 0.0bits)

PDE6A_Human  YHSPSSMEESEIIFDLLRDFQENLQTEKCIFNVMKKLCFLLQADRMSLFMY  99
PDE6B_HUMAN  LRDLCQVEESTALLELVQDMQESINMERVVFKVLRRLCTLLQADRCSLFMY  97
Consensus    *******EES*****L**D*QE****E***F*V***LC*LLQADR*SLFMY
Conservation (100% / 0%)
Sequence logo (4.3bits / 0.0bits)

PDE6A_Human  RTRNGIAELATRLFNVHKDAVLEDCLVMPDQEIVFPLDMGIVGHVAHSKKI  150
PDE6B_HUMAN  RQRNGVAELATRLFSVQPDSVLEDCLVPPDSEIVFPLDIGVVGHVAQTKKM  148
Consensus    R*RNG*AELATRLF*V**D*VLEDCLV*PD*EIVFPLD*G*VGHVA**KK*
Conservation (100% / 0%)
Sequence logo (4.3bits / 0.0bits)

PDE6A_Human  ANVPNTEEDEHFCDFVDILTEYKTKNILASPIMNGKDVVAIIMAVNKVDGS  201
PDE6B_HUMAN  VNVEDVAECPHFSSFADELTDYKTKNMLATPIMNGKDVVAVIMAVNKLNGP  199
Consensus    *NV****E**HF**F*D*LT*YKTKN*LA*PIMNGKDVVA*IMAVNK**G*
Conservation (100% / 0%)
Sequence logo (4.3bits / 0.0bits)

PDE6A_Human  HFTKRDEEILLKYLNFANLIMKVYHLSYLHNCETRRGQILLWSGSKVFEEL  252
PDE6B_HUMAN  FFTSEDEDVFLKYLNFATLYLKIYHLSYLHNCETRRGQVLLWSANKVFEEL  250
Consensus    *FT**DE***LKYLNFA*L**K*YHLSYLHNCETRRGQ*LLWS**KVFEEL
Conservation (100% / 0%)
Sequence logo (4.3bits / 0.0bits)

PDE6A_Human  TDIERQFHKALYTVRAFLNCDRYSVGLLDMTKQKEFFDVWPVLMGEVPPYS  303
PDE6B_HUMAN  TDIERQFHKAFYTVRAYLNCERYSVGLLDMTKEKEFFDVWSVLMGESQPYS  301
Consensus    TDIERQFHKA*YTVRA*LNC*RYSVGLLDMTK*KEFFDVW*VLMGE**PYS
Conservation (100% / 0%)
Sequence logo (4.3bits / 0.0bits)

PDE6A_Human  GPRTPDGREINFYKVIDYILHGKEDIKVIPNPPPDHWALVSGLPAYVAQNG  354
PDE6B_HUMAN  GPRTPDGREIVFYKVIDYVLHGKEEIKVIPTPSADHWALASGLPSYVAESG  352
Consensus    GPRTPDGREI*FYKVIDY*LHGKE*IKVIP*P**DHWAL*SGLP*YVA**G
Conservation (100% / 0%)
Sequence logo (4.3bits / 0.0bits)

PDE6A_Human  LICNIMNAPAEDFFAFQKEPLDESGWMIKNVLSMPIVNKKEEIVGVATFYN  405
PDE6B_HUMAN  FICNIMNASADEMFKFQEGALDDSGWLIKNVLSMPIVNKKEEIVGVATFYN  403
Consensus    *ICNIMNA*A***F*FQ***LD*SGW*IKNVLSMPIVNKKEEIVGVATFYN
Conservation (100% / 0%)
Sequence logo (4.3bits / 0.0bits)

PDE6A_Human  RKDGKPFDEMDETLMESLTQFLGWSVLNPDTYESMNKLENRKDIFQDIVKY  456
PDE6B_HUMAN  RKDGKPFDEQDEVLMESLTQFLGWSVMNTDTYDKMNKLENRKDIAQDMVLY  454
Consensus    RKDGKPFDE*DE*LMESLTQFLGWSV*N*DTY**MNKLENRKDI*QD*V*Y
Conservation (100% / 0%)
Sequence logo (4.3bits / 0.0bits)
```

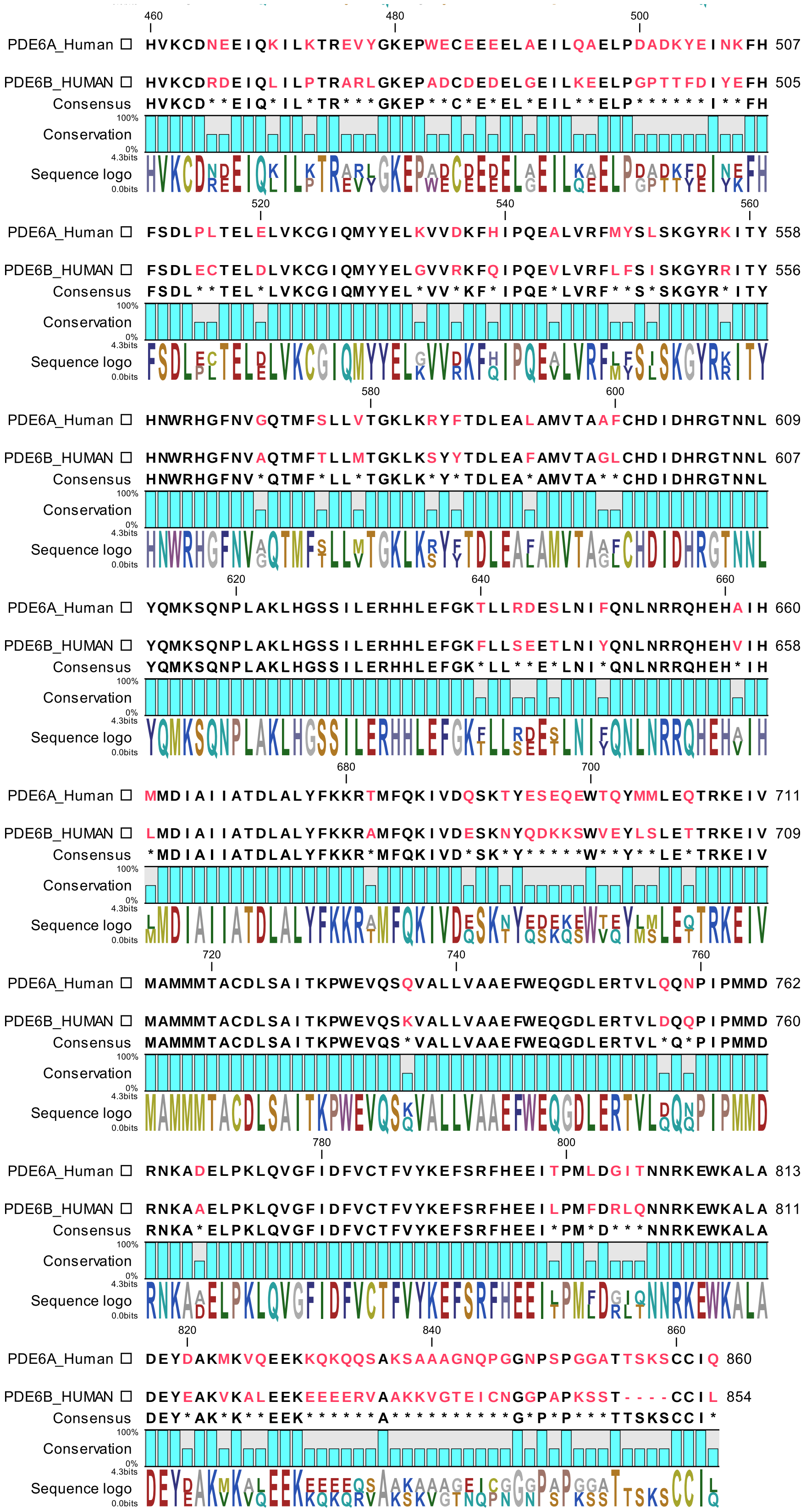

Supplementary Figure 1: Sequence alignment of Human PDE6α and PDE6β chains using CLC workbench. Identical residues among both the chains are shown in black color while consensus residues, conservation bars plots (0-100) and sequence logos are also given at the bottom to highlight conservation in both the chain's amino acid sequence.

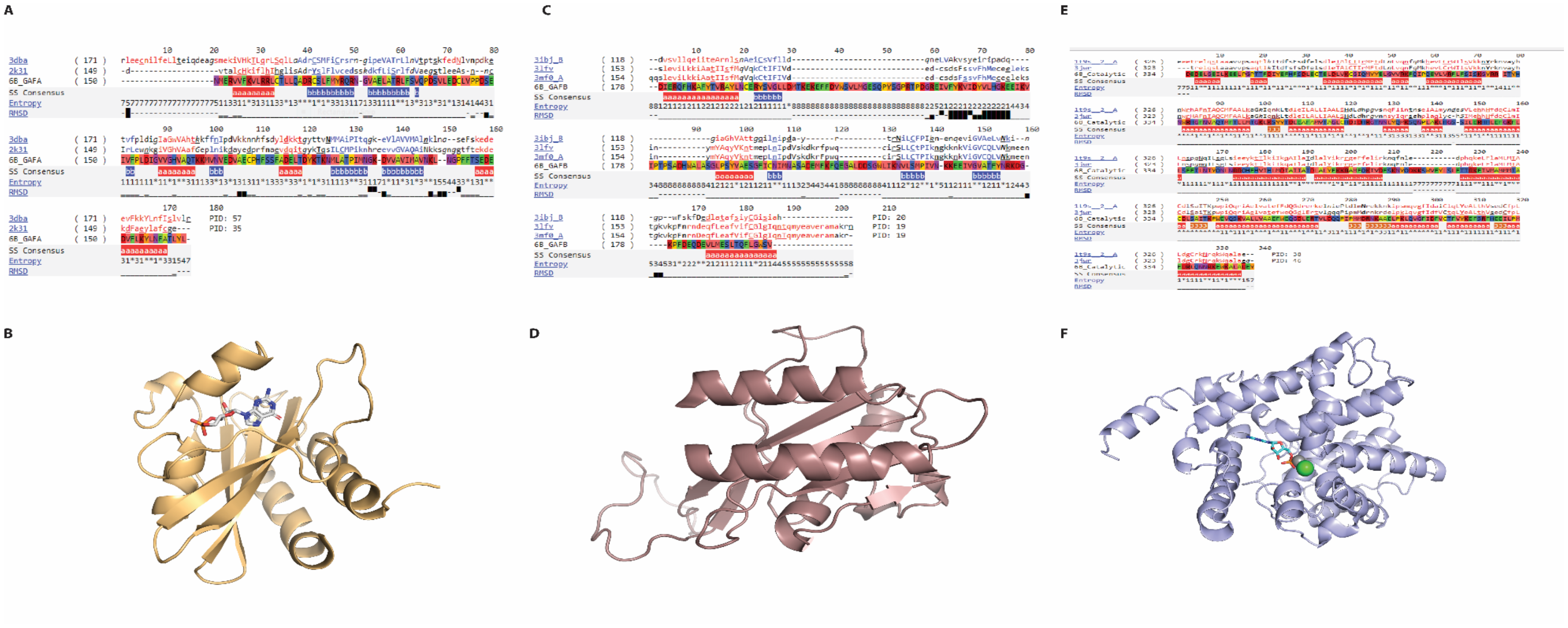

Supplementary Figure 2:Fugue Alignment and homology model of Gaf-A (A, B), Gaf-B (C, D) and catalytic domain (E, F) of PDE6α chain.

Supplementary figure 3

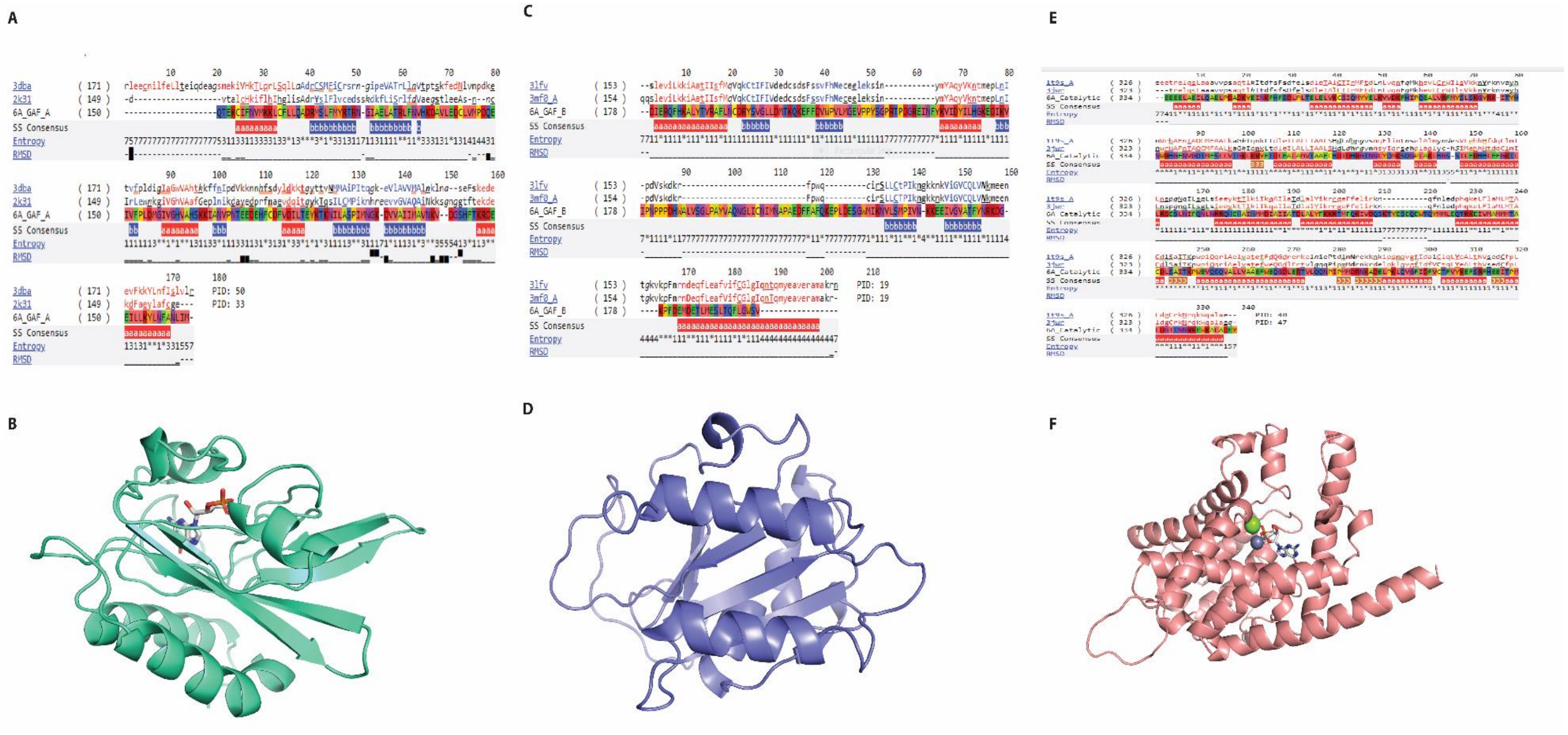

Supplementary Figure 3: Fugue Alignment and homology model of Gaf-A (A, B), Gaf-B (C, D) and catalytic domain (E, F) of PDE6β chain.

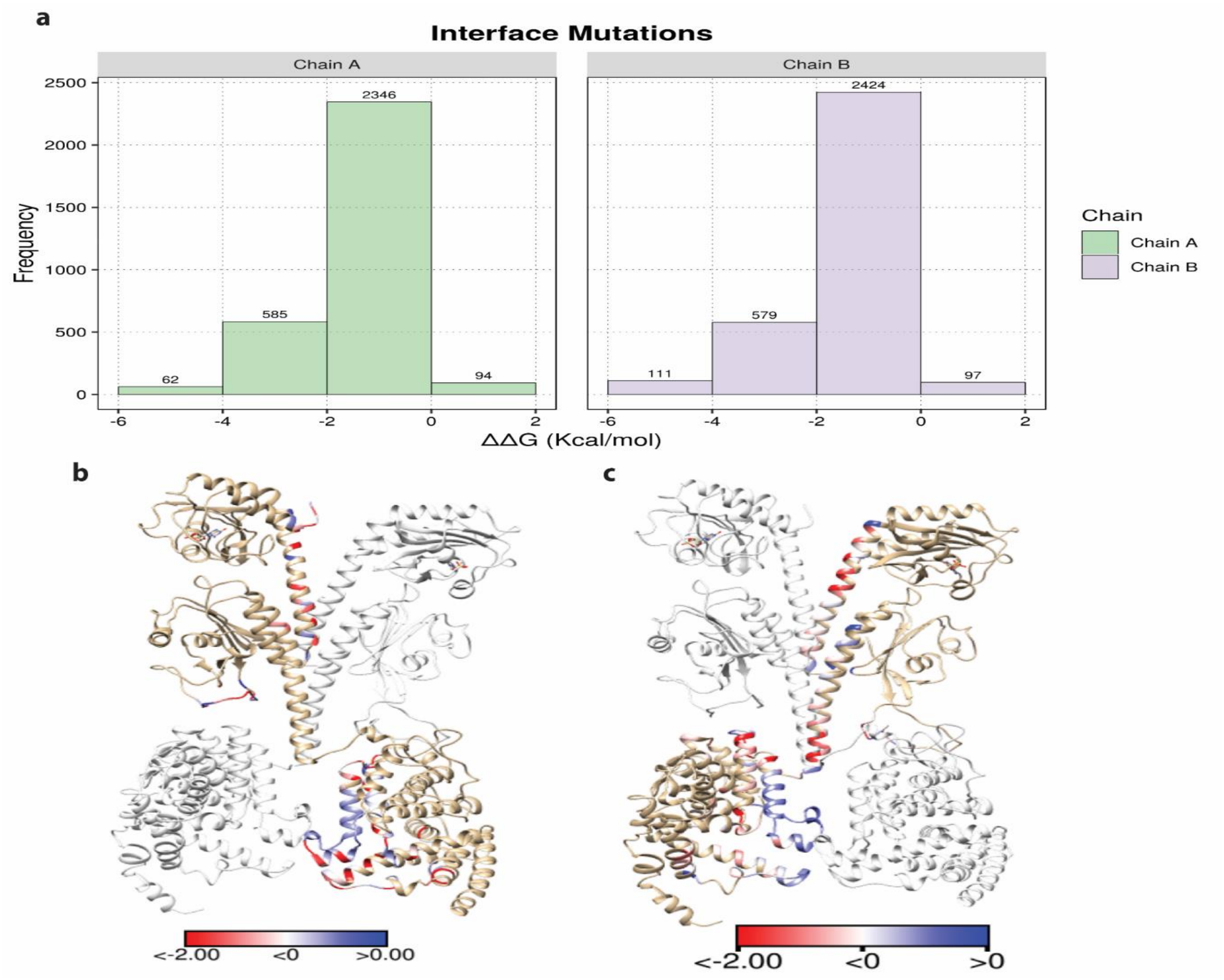

Supplementary Figure 4: a) Frequency distribution graphs illustrating the distribution of effects of saturation mutagenesis of dimeric interface residues of PDE6 αβ in terms of ΔΔG values predicted through mCSM-PPI b) Heatmap of the mCSM-PPI predicted changes upon mutation in residues present at heterodimeric interface of PDE6αβ. Energy changes calculated by mCSM-PPI are depicted as a colored gradient scale that starts from blue and turns white to red indicating the average effect from stabilizing (>0.00 kcal/mol) shown in blue to slightly destabilizing (≥-2.0 kcal/mol) shown as white to highly destabilizing (<-2.00 kcal/mol) as red.
